# Genetic diversity and molecular epidemiology of multidrug-resistant *Mycobacterium tuberculosis* in Minas Gerais State, Brazil

**DOI:** 10.1186/s12879-015-1057-y

**Published:** 2015-08-01

**Authors:** Nayanne Gama Teixeira Dantas, Phillip Noel Suffys, Wânia da Silva Carvalho, Harrison Magdinier Gomes, Isabela Neves de Almeida, Lida Jouca de Assis, Claudio José Augusto, Michel Kireopori Gomgnimbou, Guislaine Refregier, Christophe Sola, Silvana Spíndola de Miranda

**Affiliations:** Post-Graduate Program in Infectious Diseases and Tropical Medicine, Department of Internal medicine, Faculty of Medicine, Federal University of Minas Gerais, Belo Horizonte, Brazil; Laboratory of Molecular Biology Applied to Mycobacteria, Oswaldo Cruz Institute, FIOCRUZ, Rio de Janeiro, Brazil; Laboratory of Molecular Biology and Public Health, Department of Social Pharmacy, Faculty of Pharmacy, Federal University of Minas Gerais, Belo Horizonte, Brazil; Ezequiel Dias Foundation, Belo Horizonte, Brazil; Institut for Integrative Cell Biology, I2BC, UMR9198 CEA-CNRS-UPSaclay, Orsay, France; Centre Muraz, Bobo-Dioulasso, Burkina Faso

**Keywords:** MDR-TB, Molecular epidemiology, Spoligotyping, MIRU-VNTR, IS*6110*-RFLP, Minas Gerais, Brazil

## Abstract

**Background:**

We aimed to characterize the genetic diversity of drug-resistant *Mycobacterium tuberculosis* (*MTb*) clinical isolates and investigate the molecular epidemiology of multidrug-resistant (MDR) tuberculosis from Minas Gerais State, Brazil.

**Methods:**

One hundred and four *MTb* clinical isolates were assessed by IS*6110*-RFLP, 24-locus mycobacterial interspersed repetitive units variable-number tandem repeats (MIRU-VNTR), TB-SPRINT (simultaneous spoligotyping and rifampicin-isoniazid drug-resistance mutation analysis) and 3R-SNP-typing (analysis of single-nucleotide polymorphisms in the genes involved in replication, recombination and repair functions).

**Results:**

Fifty-seven different IS*6110*-RFLP patterns were found, among which 50 had unique patterns and 17 were grouped into seven clusters. The discriminatory index (Hunter and Gaston, HGDI) for RFLP was 0.9937. Ninety-nine different MIRU-VNTR patterns were found, 95 of which had unique patterns and nine isolates were grouped into four clusters. The major allelic diversity index in the MIRU-VNTR loci ranged from 0.6568 to 0.7789. The global HGDI for MIRU-VNTR was 0.9991. Thirty-two different spoligotyping profiles were found: 16 unique patterns (*n* = 16) and 16 clustered profiles (*n* = 88). The HGDI for spoligotyping was 0.9009. The spoligotyped clinical isolates were phylogenetically classified into Latin-American Mediterranean (66.34 %), T (14.42 %), Haarlem (5.76 %), X (1.92 %), S (1.92 %) and U (unknown profile; 8.65 %). Among the U isolates, 77.8 % were classified further by 3R-SNP-typing as 44.5 % Haarlem and 33.3 % LAM, while the 22.2 % remaining were not classified. Among the 104 clinical isolates, 86 were identified by TB-SPRINT as MDR, 12 were resistant to rifampicin only, one was resistant to isoniazid only, three were susceptible to both drugs, and two were not successfully amplified by PCR. A total of 42, 28 and eight isolates had mutations in *rpoB* positions 531, 526 and 516, respectively. Correlating the cluster analysis with the patient data did not suggest recent transmission of MDR-TB.

**Conclusions:**

Although our results do not suggest strong transmission of MDR-TB in Minas Gerais (using a classical 100 % MDR-TB identical isolates cluster definition), use of a smoother cluster definition (>85 % similarity) does not allow us to fully eliminate this possibility; hence, around 20–30 % of the isolates we analyzed might be MDR-TB transmission cases.

## Background

Multidrug-resistant (MDR) tuberculosis (TB) is an increasingly serious global public health threat that requires robust, efficient and quick actions to improve the control and spread of drug-resistant clinical isolates. TB isolates with resistance to isoniazid (INH) and rifampin (RIF), defined as MDR-TB, are prone to sequential accumulation of mutations in the target genes that confer resistance to them [1].

Brazil ranks sixteenth among the world’s 22 countries with high TB burdens; here, the TB prevalence is 92,000 cases, the incidence rate is 35.4 per 100,000 per year, and the mortality rate is 4.9 per 100,000 of the population according to World Health Organization estimates [2]. Minas Gerais State has the fourth lowest TB incidence (17.9/100 000/year) in Brazil [3]. Although the current prevalence of primary MDR-TB is relatively low in Brazil, it has potential to become a major public health issue, because resistance to more than one drug has been shown to be strongly associated with household contact, as suggested in a recent study conducted in Amazonia [4].

*Mycobacterium tuberculosis* complex (MTBC) genotyping methods have been widely used for investigating epidemics involving MDR-TB [5]. These methods help to define the recent transmission factors for MDR-TB isolates and enable better control programs to be initiated to avoid MDR-TB expansion at local or global population levels.

Among the various genetic markers available for studying genetic polymorphism in drug-resistant *M. tuberculosis* (*MTb*), restriction fragment length polymorphism (RFLP) analysis of the IS*6110* insertion sequence is a “gold standard” for MTBC typing [5–7]; this method has been used widely to identify and investigate TB transmission and re-infection rates, and was used at the end of the 1990s to identify cross-contamination in laboratories [7].

However, the IS*6110*-RFLP technique has many disadvantages in routine practice: it is laborious, requires trained staff, replies on microgram quantities of purified DNA, and has poor discriminatory power when applied to isolates with low IS*6110* copy numbers [5, 6, 8]. The method based on mycobacterial interspersed repetitive units variable-number tandem repeats (MIRU-VNTR) has progressively replaced IS*6110*-RFLP. MIRU-VNTR, a very powerful technique, provides adequate discrimination between *MTb* clinical isolates and is comparable to IS*6110*-RFLP in terms of its accuracy for estimating TB outbreaks and for use in phylogenetic investigations [5, 8–13]. MIRU-VNTR analysis also generates readily comparable numerical values, a useful feature for interlaboratory studies; hence, over the last 10 years it has come into standard use in TB research [9], but may progressively be replaced by whole genome sequencing (WGS) [14].

Spacer oligonucleotide typing (spoligotyping), another well-established (1996) technique, is based on the polymorphisms found in the clustered regularly interspersed short palindromic repeats (CRISPR) of *MTb*. Spoligotyping detects the presence or absence of 43 spacer sequences in the CRISPR region of *MTb* [5, 15, 16]. Spoligotyping data can be expressed in an octal or binary format. Additionally, spoligotyping requires small amounts of crude or purified DNA and its results are as portable as MIRU-VNTR, making data from it easily shared between laboratories. Spoligotyping has provided a lot of highly informative results on the phylogeographic distribution of genotypic diversity in *MTb*. Furthermore, spoligotyping in combination with MIRU-VNTR has excellent discriminatory power for cluster analysis of tubercle bacilli genomics, making it a valuable tool for the epidemiology and evolutionary biology of *MTb* [17–19]. However, because the discriminatory power of spoligotyping is generally inferior to that of IS*6110*-based RFLP, it cannot be used alone for molecular epidemiology studies [20–22].

The use of single-nucleotide polymorphisms (SNPs) as markers of genetic variation for phylogenetic analysis has been described in many studies [23–28]. Because SNPs offer important advantages for high-throughput analyses, they are the markers of choice in genetics research, likewise are regions where deletions in the genome occur [29]. Despite MTBC structural genes exhibiting very low levels of polymorphism among strains [30–33], higher polymorphism levels were found recently in several genes among which were those involved in replication, recombination and repair functions (3R genes) [34]. Indeed, the high-throughput 3R-SNP-typing method is able to classify undefined spoligotype signatures making it an efficient, easy to use tool for evolutionary studies on MTBC clinical isolates [23].

For genetic characterization of TB drug resistance, molecular detection tests currently search for known mutations in different TB-specific target genes [33]. For MDR-TB, the most frequent mutations associated with RIF and INH resistance can be assessed by sequencing, line-probe assays or other methods [28, 29]. RIF resistance is mainly (95 %) caused by the 81-bp rifampin resistance-determining region (RRDR) of the *rpoB* gene [35, 36]. INH resistance is often caused by mutations in *katG* (codon 315), *inhA* (positions −15 and −8 in the *inhA* promoter sequence), and in other genes [35, 37]. Phenotypic TB culture-based drug susceptibility testing (DST), however, remains the gold standard for diagnosis of MDR-TB [38]. Tuberculosis-spoligo-rifampin-isoniazid typing (TB-SPRINT), a 59-plex multiplexed microbead-based, high-throughput DNA array method, provides simultaneous spoligotyping and mutation analysis of the most common resistance-associated SNPs for RIF (*rpoB* RRDR direct and indirect coverage) and INH resistance (*katG, inhA*) [36, 38–40]. This method will soon be replaced by an improved 77-plex version (TB-SPRINT-*plus*) capable of identifying mutations in target genes conferring resistance to some second-line drugs (Molina et al., unpublished observations).

Here, we aimed to investigate the genetic profile of MDR-TB clinical isolates from Minas Gerais, a Brazilian state, using the following four molecular techniques: IS*6110*-RFLP, MIRU-VNTR, TB-SPRINT and 3R-SNPs-typing.

## Methods

### Clinical isolates and drug susceptibility testing

One hundred and four MDR-TB clinical isolates were collected from 2008 to 2013 in Minas Gerais, Brazil, each one corresponding to a unique TB patient. All the isolates were obtained by culture of respiratory samples and represent distinct local laboratories in Minas Gerais. The isolates were referred to the Ezequiel Dias Foundation (FUNED) for culture, identification and drug susceptibility testing. Samples were transferred to the Research Laboratory of Mycobacteria of the Faculty of Medicine of the Federal University of Minas Gerais (UFMG) where they now belong to the *MTb* clinical isolate collection. The samples are representative of MDR-TB in Minas Gerais. All the clinical isolates were from patients diagnosed with MDR-TB by the BACTEC™ MGIT™ 960 System [41]. Demographic data were obtained from the Information System on Diseases of Compulsory Declaration of Brazil (otherwise known as SINAN).

### Genomic DNA extraction

*MTb* genomic DNA was extracted from mycobacterial colonies subcultured on Löwenstein-Jensen (LJ) medium. One loopful of mycobacterial colonies was collected in a tube containing 500 μL of TE buffer (10 mM Tris-Cl, 1 mM EDTA) and then incubated at 80 °C for 60 min. Lysozyme 10 mg/mL (70 μL) was added to each tube, followed by incubation at 65 °C for 15 min with occasional mixing, after which 5 M NaCl (100 μL) and 10 % cetyltrimethylammonium bromide (CTAB) (100 μL) were added to each sample. After adding 70 μL of 10 % SDS and 6 μL of proteinase K (10 mg/mL) the samples were vortexed briefly and then incubated at 65 °C for 15 min. Chloroform/isoamyl alcohol (24:1 *v/v*) (700 μL) was added to each tube, and the solution was centrifuged for 20 min at 12,000 rpm at 4 °C in a microcentrifuge. The supernatant was transferred to a new 1.5 mL microcentrifuge tube, 450 μL of ice-cold isopropanol was added, and the tube was inverted 20 times to precipitate the nucleic acids. Samples were incubated overnight at −20 °C and then centrifuged at 12,000 rpm for 30 min at 4 °C in a microcentrifuge, after which the supernatant was discarded. The pellet was air-dried for 2 h and then resuspended in 60 μL of TE buffer (10 mM Tris-Cl, 1 mM EDTA).

### Genotyping methods

#### *IS6110*-RFLP

DNA samples were typed by IS*6110*-RFLP analysis in accordance with the standardized protocol described by van Embden et al. [7] and van Soolingen et al. [42]. The reference strain used was Mt 14323.

#### MIRU-VNTR

The standard 24 MIRU-VNTR loci method [18] was performed based on agarose gel electrophoresis. The simplex PCR product size was determined as previously reported [43].

#### TB-SPRINT

High-throughput TB-SPRINT was performed at the Institute of Genetics and Microbiology at the University Paris-Sud, France, on a Luminex 200™ flow cytometry device (Luminex Corp, Austin, TX) as previously described, using a microbead-based DNA array method [44–46]. The TB-SPRINT analysis was performed according to the standardized protocol recommended by Gomgnimbou et al. [37].

#### 3R-SNP typing

The 3R-SNP typing was performed as described by Abadia et al. [23]. This seven gene multiplex-PCR method uses primers designed on the dual-priming oligonucleotide principle, which has been shown to strongly increase the mutated to wild-type signal ratio [47].

### Bioinformatic cluster analysis

**Fig. 1:**
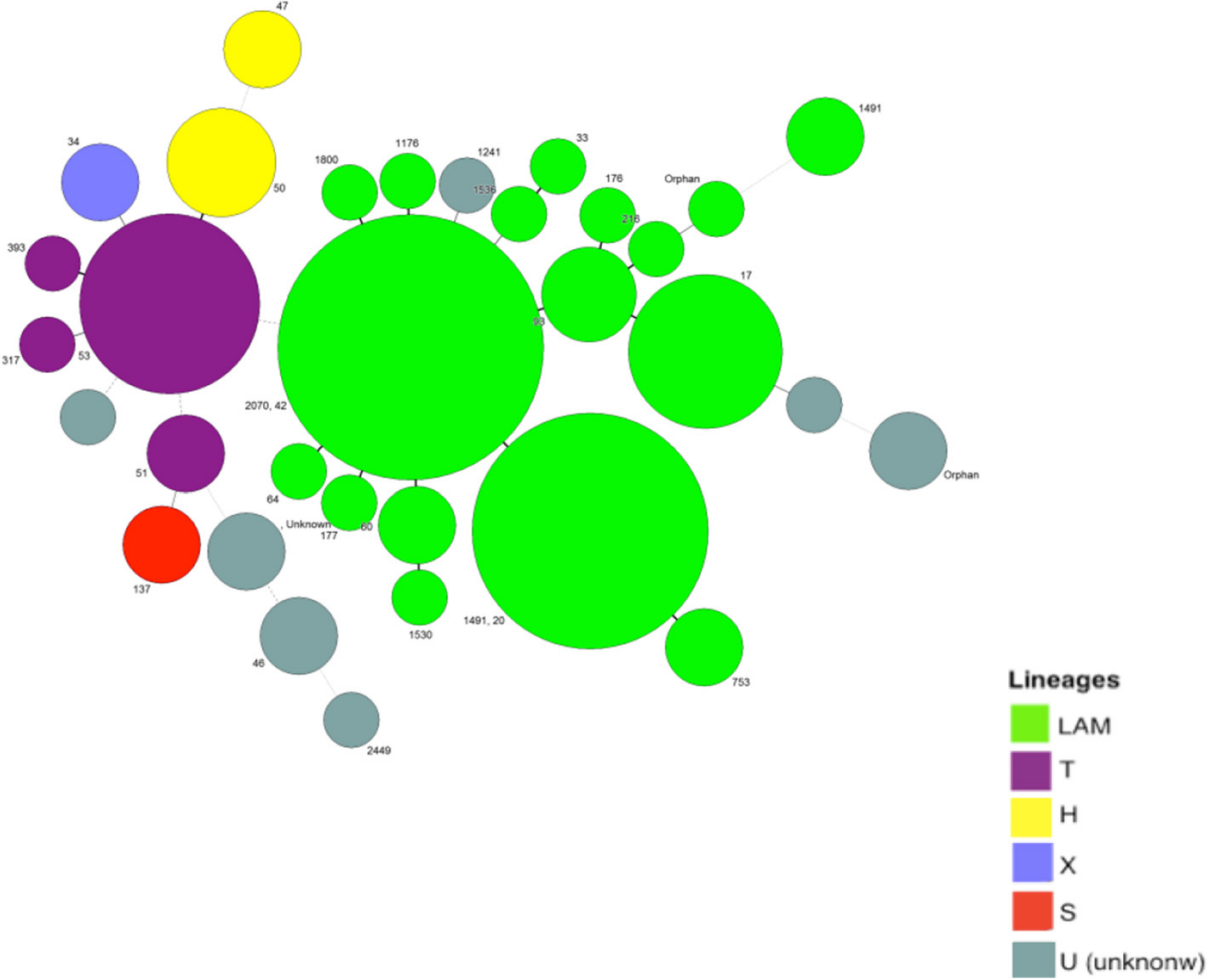
Minimum Spanning Tree (MST) obtained from the spoligotyping dataset (*n* = 104 MDR-TB isolates) identified from 2008 to 2013 in Minas Gerais, Brazil. Color code indicates major subclades found within the Lineage 4 (Euro-American), LAM, T, H, X, S and U

**Fig. 2:**
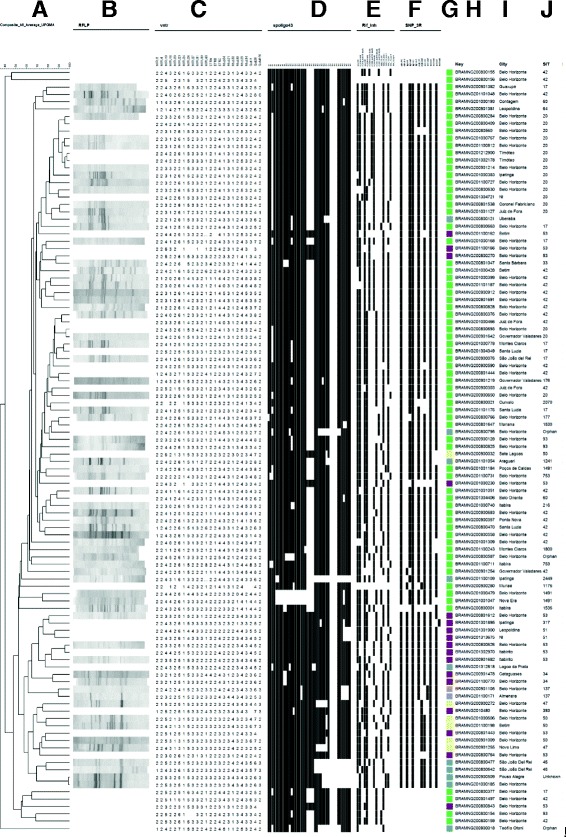
Results Matrix of the 104 studied clinical isolates. **a**: final composite dendrogram built using UPGMA using five results data set (**b**: IS*6110*-RFLP; **c**: MIRU-VNTR; **d**: spoligotyping; **e**: RIF-INH SNPs typing; **f**: 3R-SNPs-typing; **g**: subclade color code of Fig. 1; **h**: (Key) Unique Clinical isolate number; **i**: city of isolation; **j**: (SIT) Spoligo-international-type label

All results (except IS*6110*-RFLP) were entered into Excel® spreadsheets, and then transferred to BioNumerics™ software version 6.6 (Applied Maths, Sint-Martens-Latem, Belgium). IS*6110* RFLP fingerprints were digitalized and compared using the Dice coefficient and the unweighted-pair group method using average linkage (UPGMA) according to the manufacturer’s instructions [44]. MIRU-VNTR data were analyzed using the categorical coefficient and UPGMA [45]. TB-SPRINT and 3R-SNP-typing data were analyzed in BioNumerics™ using the Jaccard index and UPGMA [37]. Spoligotyping data were also analyzed using the minimum spanning tree (MST) method, as shown in Fig. 1. A composite data set for the four methods mentioned above and a composite dendrogram were also built (Fig. 2) [46]. Cluster definition was based on identical patterns using the above four methods (tighter definition) or by setting the percentage similarity at >85 % (smoother definition) [48, 49]. The recent transmission index was determined by computing the n and (n minus 1) index [50, 51].

Spoligotyping patterns were assigned a SIT (spoligotyping-international-type) using the SITVITWEB website (http://www.pasteur-guadeloupe.fr:8081/SITVIT_ONLINE/) [19]. MIRU-VNTR data were analyzed using the MIRU-VNTRplus website (http://www.miru-vntrplus.org/).

The VNTR allelic diversity index (h) was used to evaluate the allelic diversity of the various VNTR loci. The value of h was calculated using the formula described by Selander et al. [52]. The discriminatory power of each typing method was also computed and compared by the Hunter-Gaston discriminatory index (HGDI) [53, 54]. The HGDI was calculated using the discriminatory power calculator available at http://insilico.ehu.es/mini_tools/discriminatory_power/index.php.

To correlate the molecular and patient data, an analysis of basic demographic patient data (city and address) and familiar data (mother’s name) was performed.

### Ethics statement

The study was approved by the Ethics Committee of the Federal University of Minas Gerais (number 122.941; CAAE 06611912.8.0000.5149). Isolates from this study were obtained by culturing stock clinical isolates.

## Results and discussion

This is the first molecular characterization of MDR-TB clinical isolates from Minas Gerais State. The study is based on 104 clinical isolates obtained from patients in Minas Gerais State with MDR-TB-positive cultures between 2008 and 2013. The isolates, from 46 cities in the State of Minas Gerais, are considered to be representative of the MDR-TB strains in this region of Brazil. The mean age of the patients was 43.24 years and the male-to-female ratio was 1.66:1. Drug susceptibility testing (BACTEC™ MGIT™ 960 System method) showed all of 104 isolates were resistant to rifampin and isoniazid at least.

### IS*6110*-RFLP typing

The IS*6110* copy number from each isolate was assessed from the number of bands hybridizing with the probe. The 104 clinical isolates were typed and a total of 67 fingerprint patterns were obtained (65 %). The majority of them (94.03 %) had multiple IS*6110* copies (7–15). This high degree of IS*6110* polymorphism is in accordance with the results observed in drug-susceptible clinical isolates and suggests a recent low rate of MDR-TB transmission [55–60]. That we observed a low frequency of isolates with low IS*6110* copy numbers (5.97 %) demonstrates the excellent discriminatory power of IS*6110*-RFLP in our setting. Thirty-seven isolates (35 %) lacked IS*6110*-RFLP profiles, probably resulting from poor DNA quantity. Fifty-seven different IS*6110* RFLP patterns were identified, 50 of which were unique, and 17 isolates were found in seven clusters (HGDI = 0.9937).

### MIRU-VNTR

The 104 isolates were successfully typed and 99 different MIRU-VNTR patterns were found. Among these patterns, 95 were unique, and nine isolates belonged to four clusters (HGDI = 0.9991). Lineage signature was performed by MIRU-VNTRplus best-match labeling using 24 global MIRU-VNTR loci and the following six main lineages/sublineages were observed: Cameroon (3.85 %), CAS/Delhi (0.96 %), Haarlem (17.3 %), LAM (73.08 %), S (1.92 %) and T2-Uganda (2.88 %). The high HGDI of the 24-MIRU-VNTR confirms the importance of using this technique, either in association with RFLP or with spoligotyping [17, 18, 51, 60–62].

The allelic diversity of each MIRU-VNTR locus in our setting was evaluated and classified into highly (HGDI >0.6), moderately (0.6> HGDI <0.3) or poorly discriminative (HGDI <0.3) [52], as summarized in Table 1. The highest allelic diversity indexes were for Qub 26, Mtub 04, MIRU 26, MIRU 16, Qub 11, and MIRU 10. The allelic diversity index was low (*h* ≤ 0.3) for five of the 24 loci. As supported by this study and others, partial MIRU-VNTR genotyping could be sufficient to define epi-linked clusters after first-line and high-throughput spoligotyping [63]. Ali et al. described seven loci with the highest discriminatory level that could be used preferentially to investigate possible transmission events [61].

**Table 1:** Allelic diversity of each MIRU-VNTR locus and their discriminatory power

Locus	Allelic diversity	Allele’s quantity	Discriminatory
	(h) ^a^		Power^b^
Qub 26	0.7789	7	High
Mtub 04	0.7702	6	High
MIRU 26	0.7679	6	High
MIRU 16	0.7422	6	High
Qub 11	0.6975	7	High
MIRU 10	0.6568	6	High
Mtub34	0.5917	5	Moderate
MIRU 23	0.5830	6	Moderate
MIRU 40	0.5791	7	Moderate
MIRU 27	0.5624	4	Moderate
Mtub 39	0.4901	5	Moderate
Mtub 30	0.4462	4	Moderate
Qub 4156	0.4360	4	Moderate
Mtub 21	0.4199	4	Moderate
Mtub 29	0.4154	4	Moderate
MIRU 31	0.4147	6	Moderate
ETRC	0.3482	4	Moderate
ETRA	0.3308	4	Moderate
ETRB	0.3278	2	Moderate
MIRU 39	0.2678	3	Low
MIRU 2	0.1755	2	Low
MIRU 20	0.1755	2	Low
MIRU 4	0.1113	3	Low
MIRU 24	0.0194	2	Low

*n* = 104 *MTb* isolates

^a^Calculated as described by Selander et al. [52]

^b^Discriminatory power: high (h > 0.6), moderate (0.3 < h > 0.6) and low (h ≤ 0.3)

### TB-SPRINT typing

All the clinical isolates were successfully typed by spoligotyping and were classified phylogenetically into five lineages and 11 sublineages, as shown in Table 2. Thirty-two different spoligotyping patterns were found; 16 of them had unique patterns, and 88 isolates were grouped into 16 clusters (HGDI = 0.9009). A minimum spanning tree (MST) was built (Fig. 1). The single lineage is lineage four (Euro-American) with a majority of LAM, T, H and a minority of S and *X*2. The two major sublineages (LAM: *n* = 69 and T: *n* = 15) are in central positions of the MST (Fig. 1). They show tight links between patterns and represent the major proportion of the clinical isolates in the Minas Gerais State (*n* = 84/104 or 80.76 % of the clinical isolates). The continuous transmission of MTB in certain settings is strongly affected by the prevailing population structure of tubercle bacilli [63], which induces the predominance of a homogeneous group, such as the LAM family in South America and similarly for the Beijing family in Asia [19, 57, 64–66]. LAM, T and H clinical isolates are more likely to become MDR in Brazil [67, 68].

**Table 2:** Phylogenetical classification of multidrug-resistant *Mycobacterium tuberculosis* (*n* = 104) by TB-SPRINT method

Lineage	n (%)	Sublineage	n (%)	SIT number	n (%)
LAM	69 (66.4 %)				
		LAM 1	19		
				SIT 20	89.5 %
				SIT 753	10.5 %
		LAM 2	8		
				SIT 17	100 %
		LAM 3	4		
				SIT 1491	75 %
				SIT 33	25 %
		LAM4	3		
				SIT 60	66.7 %
				SIT 1530	33.3 %
		LAM 5	5		
				SIT 93	60 %
				SIT 216	20 %
				ORPHAN	20 %
		LAM 6	2		
				SIT 64	50 %
				SIT 176	50 %
		LAM 9	28		
				SIT 1800	3.6 %
				SIT 177	3.6 %
				SIT 2070	3.6 %
				SIT 1176	3.6 %
				SIT 1536	3.6 %
				SIT 42	82 %
T	15 (14.4 %)				
		T1	14		
				SIT 53	70.5 %
				SIT 73	5.9 %
				SIT 317	5.9 %
				SIT 393	5.9 %
				SIT 51	11.8 %
		T2	1		
				SIT 317	100 %
Haarlem	6 (5.8 %)				
		H1	2		
				SIT 47	100 %
		H3	4		
				SIT 50	100 %
X	2 (1.9 %)				
S	2 (1.9 %)				
				SIT 34	100 %
Unknown	9 (8.7 %)				
Orphan	1 (0.9 %)				

A further characterization of the RIF-INH typing scheme (16-plex) was performed for 102 of 104 (98.7 %) isolates, either on the 81 base-pair RRDR for RIF or in *inhA and katG* for INH. For two isolates, none of the positions tested were amplifiable. For the isolates typed at the RRDR locus, 98 (96.07 %) had mutated genotypes, among which 78 (76.4 % of total) carried defined targeted mutations conferring RIF resistance. However, 20 isolates with undefined RIF mutations were detected and four samples had no mutations. A total of 42, 28 and eight isolates had mutations at *rpoB* positions 531 (38 samples *rpoB* 531TTG and four samples *rpoB* 531TGG), 526 (19 samples *rpoB* 526GAC and nine samples *rpoB* 526TAC) and 516 (eight samples *rpoB* 516GTC), respectively, and only one of these samples carried mutations in the three *rpoB* positions. Out of 102 isolates typed at loci known to be involved in INH resistance, 87 (85.29 %) had a mutated genotype at either *katG*315 or within the inhA promoter. Among them, 82 (94.2 %) carried the following defined mutations conferring INH resistance: 54 samples harbored *katG* 315ACC (65.85 %), four samples *katG* 315AAC (4.87 %), nine samples *inhA*-15 (10.97 %), 14 samples harbored a double *katG* 315ACC + *inhA*-15 mutation and one sample harbored a double *katG* 315AAC + *inhA*-15 mutation. These results are in accordance with other first-line TB drug resistance studies in Brazil [67, 69, 70]. Altogether, out of 102 isolates typed for both loci, 86 were resistant to both drugs (INH and RIF). Forty-five different RIF-INH profiles were obtained for the isolates and 25 of them had a unique profile, while 17 profiles clustered into 72 isolates (HGDI = 0.9561).

### 3R-SNP-typing

For some spoligotyping patterns (*n* = 9), it was not possible to assign their lineages/sublineages. To assign such isolates (called “U” isolates in SpolDB4 and/or SITVITWEB), a 3R-SNP scheme was used successfully to solve 77.8 % of the cases [23]. The patterns found were Haarlem (*n* = 4) and LAM (*n* = 3), and these signatures were confirmed by MIRU-VNTR typing. Only two samples remained unclassified.

Out of 104 isolates, the 3R-SNP-typing method allowed us to find mutations in 94 (90.38 %) isolates associated with four specific genotype families: LAM (*n* = 67), Haarlem (*n* = 12), X (*n* = 2), T2 (*n* = 1) and an unknown lineage (*n* = 12). The ten isolates remaining (9.62 %) did not amplify successfully. Use of the 3R-SNP-based method helped to clarify the infra-specific taxonomy of our sampling, thus improving our confidence in the evolutionary analysis of our data [23, 71].

### Comparison of the discriminatory powers of the genotyping methods

The discriminatory powers of the IS*6110*-RFLP, MIRU-VNTR, TB-SPRINT (spoligotyping and RIF-INH-typing) and 3R-SNP methodologies are shown in Table 3. Use of a combination of different techniques is important for improved epidemiological and phylogeographical interpretation of molecular results [17, 18, 51, 61, 62]. MIRU-VNTR has the highest discriminatory power followed by IS*6110*-RFLP and TB-SPRINT. 3R-SNP-typing has a lower discriminatory power because only seven SNPs were used in the current format.

**Table 3:** Discriminatory index of IS*6110*-RFLP, MIRU-VNTR, TB-SPRINT (Spoligotyping and RIF-INH typing), 3R-SNPs

Typing method	Number	No. of different profiles	No. of uniques profiles	No. of Clusters	HGDI^a^
IS6110-RFLP	67	57	50	7	0.9937
MIRU-VNTR	104	99	95	4	0.9991
RIF-INH-typing	102	47	25	17	0.9561
Spoligotyping	104	32	16	16	0.9009
3R-SNPs	96	13	5	8	0.5466

^a^Calculated as described by Selander et al. [52]

### Molecular epidemiology in the Minas Gerais State

Among the 104 clinical isolates, 71 displayed a low similarity index (<85 %) and 33 a high similarity index (>85 %). Twelve clusters without any obvious epidemiological link were observed. According to the (n minus 1) or n Recent Transmission Index [50, 51] definition (where there is the choice to diminish or not to diminish all the clusters by one index case) and using the smooth cluster definition (>85 %), the maximum transmission rate of MDR-TB in Minas Gerais State would be (33 minus 12/104), or 20 % using the (n minus 1) method, and 33/104 or 31 % using the n method. However, if we assume a 100 % identity cluster definition, no 100 % identity cluster was found, which suggests that no cases of MDR-TB transmission occurred in Minas Gerais State. Our results clearly point to an extended classical “stone in the pound” epidemiological analysis of the 12 suspected clusters, for which MDR-TB transmission remains likely [72].

Our results are possibly explained by the fact that MDR-TB transmission in Minas Gerais is individually acquired (i.e., there are no primary MDR-TB cases). An alternative explanation is that many cases of MDR-TB were missed, but this seems unlikely because the sampling is representative of MDR-TB cases in Minas Gerais. The situation for Minas Gerais differs from that of other Brazilian studies [67, 73] in that the other studies did not use all the techniques used herein, which may have increased the discriminatory power of our analysis. This discordance could also be explained by the global differences in TB prevalence between different regions of Brazil.

When looking more closely at the geographical origin of the samples from Minas Gerais, we could identify only one factor that made us suspicious of an epidemiological link (in eight patients from the same city). Our results suggest that clustered genotypes indicative of recent MDR-TB transmission should be interpreted with caution, unless direct evidence of epidemiological links between clustered cases can be demonstrated [74].

Despite the low number of samples, this collection of MTBC isolates is likely to be representative of the confirmed MDR-TB cases in Minas Gerais State. One limitation of the present study, however, is the absence of clinical epidemiological data. Also, contact tracing for individual patients could not be performed.

Future long-term studies are necessary to identify the possible risk factors for the emergence of drug resistance and/or treatment failure. Additionally, longitudinal studies in regions of Brazil with a high incidence of MDR-TB are now urgently needed.

## Conclusions

To sum up, use of four different discriminant genotyping techniques (*IS6110*-RFLP, MIRU-VNTR, TB-SPRINT and 3R-SNP-typing) provided useful data for phylogenetic evaluation and fine taxonomic characterization of MDR-TB clinical isolates from Minas Gerais State, Brazil. The most common MDR-TB isolates belonged to the LAM lineage and approximately two thirds of them did not provide evidence for recent transmission of MDR-TB. Our data indicate that MDR-TB in Minas Gerais State is caused by clinical isolates that were not transmitted in recent years or that the outbreak is driven by individually acquired resistance and endogenous reactivation. This situation contrasts with the findings from other Brazilian studies, which all reported a high transmission rate for MDR-TB. Such an important issue requires locally-adapted solutions and state-specific control measures in Brazil. Continuous surveillance of MDR-TB transmission could be improved by introduction of new diagnostic tools and epidemiological research using WGS methods.

## Abbreviations

*MTb*: *Mycobacterium tuberculosis*
MDR: Multidrug-resistant
TB: Tuberculosis
HGDI: Hunter-Gaston discriminatory index
LAM: Latin-American Mediterranean
U: Unknown profile
INH: Isoniazid
RIF: Rifampin
MTBC: *Mycobacterium tuberculosis* complex
RFLP: Restriction fragment length polymorphism
IS: Insertion sequence
MIRU-VNTR: Mycobacterial interspersed repetitive units variable-number tandem repeats
WGS: Whole genome sequencing
Spoligotyping: Spacer oligonucleotide typing
CRISPR: Clustered regularly interspersed short palindromic repeats
PCR: Polymerase chain reaction
RRDR: Rifampin resistance-determining region
DST: Drug susceptibility testing
SNPs: Single-nucleotide polymorphism
TB-SPRINT: Tuberculosis-spoligo-rifampin-isoniazid typing
3R: Replication, recombination and repair functions
FUNED: Ezequiel Dias Foundation
UFMG: Federal University of Minas Gerais
SINAN: Information system on diseases of compulsory declaration
LJ: Löwenstein-Jensen
DPO: Dual-priming oligonucleotide
UPGMA: Unweighted-pair group method using average linkage
MST: Minimum spanning trees
SIT: Spoligotyping-international-type
h: Allelic diversity index
RTI: Recent transmission index

## References

[1] Brossier F, Sola C, Millota G, Jarliera V, Veziris N, Sougakoff W (2014). Comparison of a Semiautomated Commercial Repetitive-Sequence-Based PCR Method with Spoligotyping, 24-Locus Mycobacterial Interspersed Repetitive-Unit–Variable-Number Tandem-Repeat Typing, and Restriction Fragment Length Polymorphism-Based Analysis of IS6110 for *Mycobacterium tuberculosis* Typing. J Clin Microbiol.

[2] World Health Organization (2013). WHO report 2013. Global tuberculosis control: surveillance, planning, financing. WHO/HTM/ TB/2013.306.

[3] Brazil. Tuberculose: casos confirmados notificados no Sistema de Informação de Agravos de Notificação (Sinan). Brasilia, Brazil: Ministério da Saúde, 2004. http://dtr2004.saude.gov.br/sinanweb/tabnet/dh?sinan/tuberculose/bases/tubercbr.def Accessed January 2015. [Portuguese]

[4] da Silva GM, Ramasawmy R, Perez-Porcuna TM, Zaranza E, Chrusciak Talhari A, Martinez-Espinosa FE, Bührer-Sékula S (2014). Primary drug resistance among pulmonary treatment-naive tuberculosis patients in Amazonas State. Brazil Int J Tuberc Lung Dis.

[5] Sougakoff W (2011). Molecular epidemiology of multidrug-resistant clinical isolates of *Mycobacterium tuberculosis*. Clin Microbiol Infect.

[6] McEvoy CR, Falmer AA, van Pittius NC G, Victor TC, van Helden PD, Warren RM (2007). The role of IS*6110* in the evolution of *Mycobacterium tuberculosis*. Tuberculosis.

[7] van Embden JD, Cave MD, Crawford JT, Dale JW, Eisenach KD, Gicquel B, Hermans P, Martin C, McAdam R, Shinnick TM (1993). Strain identification of *Mycobacterium tuberculosis* by DNA fingerprinting: recommendations for a standardized methodology. J Clin Microbiol.

[8] Sun YJ, Bellamy R, Lee ASG, Ng ST, Ravindran S, Wong SY, Locht C, Supply P, Paton NI (2004). Use of Mycobacterial Interspersed Repetitive Unit-Variable-Number Tandem Repeat Typing to examine genetic diversity of Mycobacterium tuberculosis in Singapore. J Clin Microbiol.

[9] Supply P, Mazars E, Lesjean S, Vincent V, Gicquel B, Locht C (2000). Variable human minisatellite‐like regions in the *Mycobacterium tuberculosis* genome. Mol Microbiol.

[10] Cowan LS, Mosher L, Diem L, Massey JP, Crawford JT (2002). Variable-number tandem repeat typing of Mycobacterium tuberculosis isolates with low copy numbers of IS6110 by using mycobacterial interspersed repetitive units. J Clin Microbiol.

[11] Mazars E, Lesjean S, Banuls AL, Gilbert M, Vincent V, Gicquel B, Tibayrenc M, Locht C, Supply P (2001). High-resolution minisatellite-based typing as a portable approach to global analysis of Mycobacterium tuberculosis molecular epidemiology. Proc Natl Acad Sci U S A.

[12] Sola C, Filliol I, Legrand E, Lesjean S, Locht C, Supply P, Rastogi N (2003). Genotyping of the Mycobacterium tuberculosis complex using MIRUs: association with VNTR and spoligotyping for molecular epidemiology and evolutionary genetics. Infect Genet Evol.

[13] Supply P, Lesjean S, Savine E, Kremer K, van Soolingen D, Locht C (2001). Automated high-throughput genotyping for study of global epidemiology of *Mycobacterium tuberculosis* based on mycobacterial interspersed repetitive units. J Clin Microbiol.

[14] Roetzer A, Diel R, Kohl TA, Rückert C, Nübel U, Blom J, Wirth T, Jaenicke S, Schuback S, Rüsch-Gerdes S, Suplly P, Kalinowski J, Niemann S (2013). Whole Genome Sequencing versus Traditional Genotyping for Investigation of a Mycobacterium tuberculosis Outbreak: A Longitudinal Molecular Epidemiological Study. PLoSMed.

[15] Zhang J, Abadia E, Refregier G, Tafaj S, Boschiroli ML, Guillard B, Andremont A, Ruimy R, Sola C (2010). *Mycobacterium tuberculosis* complex CRISPR genotyping: improving efficiency, throughput and discriminative power of ‘spoligotyping’ with new spacers and a microbead-based hybridization assay. J Med Microbiol.

[16] Kato‐Maeda M, Metcalfe JZ, Flores L (2011). Genotyping of *Mycobacterium tuberculosis*: application in epidemiologic studies. Future Microbiol.

[17] Sola C (2015). Clustered regularly interspersed short palindromic repeats (CRISPR) genetic diversity studies as a mean to reconstruct the evolution of the *Mycobacterium tuberculosis* complex. Tuberculosis (Edinb).

[18] Supply P, Allix C, Lesjean S, Cardoso-Oelemann M, Rusch-Gerdes S, Willery E, Savine E, de Haas P, van Deutekom H, Roring S, Bifani P, Kurepina N, Kreiswirth B, Sola C, Rastogi N, Vatin V, Gutierrez MC, Fauville M, Niemann S, Skuce R, Kremer K, Locht C, van Soolingen D (2006). Proposal for Standardization of Optimized Mycobacterial Interspersed Repetitive Unit-Variable-Number Tandem Repeat Typing of *Mycobacterium tuberculosis*. J Clin Microbiol.

[19] Demay C, Liens B, Burguière T, Hill V, Couvin D, Millet J (2012). SITVITWEB – A publicly available international multimarker database for studying *Mycobacterium tuberculosis* genetic diversity and molecular epidemiology. Infect Genet Evol.

[20] Kurepina N, Likhoshvay E, Shashkina E, Mathema B, Kremer K, van Soolingen D, Bifani P, Kreiswirth BN (2005). Targeted hybridization of IS*6110* fingerprints identifies the W-Beijing *Mycobacterium tuberculosis* clinical isolates among clinical isolates. J Clin Microbiol.

[21] Driscoll JR (2009). Spoligotyping for Molecular Epidemiology of the *Mycobacterium tuberculosis* Complex Molecular Epidemiology of Microorganisms. Methods in Molecular Biology™.

[22] Kamerbeek J, Schouls L, Kolk A, van Agterveld M, van Soolingen D, Kuijper S, Bunschoten A, Molhuizen H, Shaw R, Goyal M, van Embden J (1997). Simultaneous detection and strain differentiation of *Mycobacterium tuberculosis* for diagnosis and epidemiology. J Clin Microbiol.

[23] Abadia E, Zhang J, dos Vultos T, Ritacco V, Kremer K, Aktas E, Matsumoto T, Refregier G, Sola C (2010). Resolving lineage assignation on *Mycobacterium tuberculosis* clinical isolates classified by spoligotyping with a new high-throughput 3R SNPs based method. Infect Genet Evol.

[24] Comas I, Homolka S, Niemann S, Gagneux S (2009). Genotyping of genetically monomorphic bacteria: DNA sequencing in *Mycobacterium tuberculosis* highlights the limitations of current methodologies. PLoS One.

[25] Coll F, McNerney R, Guerra-Assuncao JA, Glynn JR, Perdigao J, Viveiros M, Portugal I, Pain A, Martin N, Clark TG (2014). A robust SNP barcode for typing *Mycobacterium tuberculosis* complex clinical isolates. Nat Commun.

[26] Homolka S, Projahn M, Feuerriegel S, Ubben T, Diel R, Nubel U, Niemann S (2012). High resolution discrimination of clinical *Mycobacterium tuberculosis* complex clinical isolates based on single nucleotide polymorphisms. PLoS One.

[27] Feuerriegel S, Koser CU, Niemann S (2014). Phylogenetic polymorphisms in antibiotic resistance genes of the *Mycobacterium tuberculosis* complex. J Antimicrob Chemother.

[28] Stucki D, Malla B, Hostettler S, Huna T, Feldmann J, Yeboah-Manu D, Borrell S, Fenner L, Comas I, Coscollà M (2012). Two new rapid SNP-typing methods for classifying *Mycobacterium tuberculosis* complex into the main phylogenetic lineages. PLoS One.

[29] Schork NJ, Fallin D, Lanchbury JS (2000). Single nucleotide polymorphisms and the future of genetic epidemiology. Clin Genet.

[30] Musser JM, Amin A, Ramaswamy S (2000). Negligible genetic diversity of *Mycobacterium tuberculosis* host immune system protein targets: evidence of limited selective pressure. Genetics.

[31] Sreevatsan S, Pan X, Stockbauer KE, Connell ND, Kreiswirth BN, Whittam TS, Musser JM (1997). Restricted structural gene polymorphism in the *Mycobacterium tuberculosis* complex indicates evolutionarily recent global dissemination. Proc Natl Acad Sci U S A.

[32] Kapur V, Whittam TS, Musser JM (1994). Is *Mycobacterium tuberculosis* 15,000 years old?. J Infect Dis.

[33] Achtman M (2008). Evolution, population structure, and phylogeography of genetically monomorphic bacterial pathogens. Annu Rev Microbiol.

[34] Dos Vultos T, Mestre O, Rauzier J, Golec M, Rastogi N, Rasolofo V, Tonjum T, Sola C, Matic I, Gicquel B (2008). Evolution and diversity of clonal bacteria: the paradigm of *Mycobacterium tuberculosis*. PLoS One.

[35] Gomgnimbou MK, Abadia E, Zhang J, Refregier G, Panaiotov S, Bachiyska E, Sola C (2012). “Spoligoriftyping”, a dual-priming-oligonucleotide-based direct-hybridization assay for tuberculosis control with a multianalyte microbead-based hybridization system. J Clin Microbiol.

[36] Heep M, Brandstätter B, Rieger U, Lehn N, Richter E, Rüsch-Gerdes S, Niemann S (2001). Frequency of rpoB mutations inside and outside the cluster I region in rifampin-resistant clinical *Mycobacterium tuberculosis* isolates. J Clin Microbiol.

[37] Gomgnimbou M, Hernandez-Neuta I, Panaiotov S, Bachyiska E, Palomino JC, Martin A, Portillo PD, Refrégier G, Sola C (2013). TB-SPRINT: TuBerculosis-SPoligo-Rifampin-IsoNiazid Typing”, a one-in all-assay technique for surveillance and control of multi-drug resistant tuberculosis on Luminex® devices J. Clin Microbiol.

[38] Ocheretina O, Escuyer VE, Mabou MM, Royal-Mardi G, Collins S, Vilbrun SC, Pape JW, Fitzgerald DW (2014). Correlation between Genotypic and Phenotypic Testing for Resistance to Rifampin in *Mycobacterium tuberculosis* Clinical Isolates in Haiti: Investigation of Cases with Discrepant Susceptibility Results. PLoS One.

[39] WANG X, Jiaoa J, Xua W, Chaib X, Lia Z, Wanga Q (2013). A simple, rapid and economic method for detecting multidrug-resistant tuberculosis. Braz J Infect Dis.

[40] Nikolayevskyy V, Balabanova Y, Simak T, Malomanova N, Fedorin I, Drobniewski F (2009). Performance of the Genotype MTBDRPlus assay in the diagnosis of tuberculosis and drug resistance in Samara. Russian Federation BMC Clin Pathol.

[41] Tortoli E, Benedetti M, Fontanelli A, Simonetti MT (2002). Evaluation of automated BACTEC MGIT 960 system for testing susceptibility of *Mycobacterium tuberculosis* to four major antituberculous drugs: comparison with the radiometric BACTEC 460 TB method and the agar plate method of proportion. J Clin Microbiol.

[42] van Soolingen D, de Haas PE, Hermans PW, van Embden JD (1994). DNA fingerprinting of *Mycobacterium tuberculosis*. Methods Enzymol.

[43] Bonura C, Gomgnimbou MK, Refrégier G, Aleo A, Fasciana T, Giammanco A, Sola C, Mammina C (2014). Molecular epidemiology of tuberculosis in Sicily, Italy: what has changed after a decade?. BMC Infect Dis.

[44] Hwang HY, Chang CY, Chang LL, Chang SF, Chang YH, Chen YJ (2003). Characterization of rifampicin-resistant *Mycobacterium tuberculosis* in Taiwan. J Med Microbiol.

[45] Roetzer A, Schuback S, Diel R, Gasau F, Ubben T, di Nauta A, Richter E, Rüsch-Gerdes S, Niemann S (2011). Evaluation of *Mycobacterium tuberculosis* typing methods in a 4-year study in Schleswig-Holstein. Northern Germany J Clin Microbiol.

[46] Varma-Basil M, Kumar S, Arora J, Angrup A, Zozio T, Banavaliker JN, Singh UB, Rastogi N, Bose M (2011). Comparison of spoligotyping, mycobacterial interspersed repetitive units typing and IS*6110*-RFLP in a study of genotypic diversity of *Mycobacterium tuberculosis* in Delhi. North India Mem Inst Oswaldo Cruz.

[47] Chun JY, Kim KJ, Hwang IT, Kim YJ, Lee DH, Lee IK, Kim JK (2007). Dual priming oligonucleotide system for the multiplex detection of respiratory viruses and SNP genotyping of CYP2C19 gene. Nucleic Acids Res.

[48] Borgdorff MW, van de Hof S, Kalisvaart N, Kremer K, van Soolingen D (2011). Influence of Sampling on clustering and Associations With Risk Factors in the Molecular Epidemiology of Tuberculosis. Am J Epidemiol.

[49] Fok A, Numata Y, Schulzer M, Fitzgerald MJ (2008). Risk factors for clustering of tuberculosis cases: a systematic review of population-based molecular epidemiology studies. Int J Tuberc Lung Dis.

[50] Alland D, Kalkut GE, Moss AR (1994). Transmission of tuberculosis in New York City. An analysis by DNA fingerprinting and conventional epidemiologic methods. N Engl J Med.

[51] Murray M, Alland D (2002). Methodological Problems in the Molecular Epidemiology of Tuberculosis. Am J Epidemiol.

[52] Selander RK, Caugant DA, Ochman H, Musser JM, Gilmour MN, Whittam TS (1986). Methods of multilocus enzyme electrophoresis for bacterial population genetics and systematics. Appl Environ Microbiol.

[53] Hunter PR, Gaston MA (1988). Numerical index of the discriminatory ability of typing systems: an application of Simpson’s index of diversity. J Clin Microbiol.

[54] Nakagawa T, Shibayama T, Uchiya K, Nikai T, Ogawa K (2009). Comparison of a Variable-Number Tandem-Repeat (VNTR) Method for Typing *Mycobacterium avium* with Mycobacterial Interspersed Repetitive-Unit–VNTR and IS*1245* Restriction Fragment Length Polymorphism Typing. J Clin Microbiol.

[55] Zheng C, Zhao E, Zhu G, Li S, Sun H, Feng Q, Luo M, Wu F, Li X, VŽronique C (2014). Suitability of IS6110-RFLP and MIRU-VNTR for Differentiating Spoligotyped Drug-Resistant Mycobacterium tuberculosis Clinical Isolates from Sichuan in China. Biomed Res Int.

[56] Chauhan A, Chauhan DS, Parashar D, Gupta P, Sharma VD, Sachan AS, Gupta R, Agarawal BM, Katoch VM (2004). DNA Fingerprinting of *Mycobacterium tuberculosis* isolates from AGRA region by IS*6110* probe. Indian J Med Microbiol.

[57] Niemann S, Sch-Gerdes S, Virarichter A (1997). IS*6110* Fingerprinting of drug-resistant *Mycobacterium tuberculosis* clinical isolates isolated in Germany during 1995. J Clin Microbiol.

[58] Hermans PWM, Messadi F, Guebrexabher H, van Soolingen D, de Haas PEW, Heersma H, de Neeling H, Ayoub A, Portaels F, Frommel D, Zribi M, van Embden JDA (1995). Analysis of the population structure of *Mycobacterium tuberculosis* in Ethiopia, Tunisia, and the Netherlands: usefulness of DNA typing for global tuberculosis epidemiology. J Infect Dis.

[59] Strassle A, Putnik J, Weber R, Fehr-Merhof A, Wust J, Pfyffer GE (1997). Molecular epidemiology of *Mycobacterium tuberculosis* clinical isolates isolated from patients in a human immunodeficiency virus cohort in Switzerland. J Clin Microbiol.

[60] Van Soolingen D, Hermans PWM, de Haas PEW, Soll DR, van Embden JDA (1991). Occurrence and stability of insertion sequences in *Mycobacterium tuberculosis* complex clinical isolates: evaluation of an insertion sequence-dependent DNA polymorphism as a tool in the epidemiology of tuberculosis. J Clin Microbiol.

[61] Ali A, Hasan Z, Tanveer M, Siddiqui AR, Ghebremichael S, Kallenius G, Hasan R (2007). Characterization of *Mycobacterium tuberculosis* Central Asian Strain 1 using mycobacterial interspersed repetitive unit genotyping. BMC Microbiol.

[62] Jonsson J, Hoffner S, Berggren I, Bruchfeld J, Ghebremichael S, Pennhag A, Groenheit R (2014). Comparison between RFLP and MIRU-VNTR Genotyping of *Mycobacterium tuberculosis* Clinical isolates Isolated in Stockholm 2009 to 2011. Plos one.

[63] Asante-Poku A, Nyaho MS, Borrell S, Comas I, Gagneux S, Yeboah-Manu D (2014). Evaluation of Customised Lineage-Specific Set of MIRU-VNTR Loci for Genotyping *Mycobacterium tuberculosis* Complex Isolates in Ghana. PlosOne.

[64] Mokrousov I (2012). The quiet and controversial: ural family of *Mycobacterium tuberculosis*. Infect Genet Evol.

[65] Millet J, Baboolal S, Streit E, Akpaka PE, Rastogi N (2014). A First Assessment of *Mycobacterium tuberculosis* Genetic Diversity and Drug-Resistance Patterns in Twelve Caribbean Territories. BioMed Res Int.

[66] Miranda SS, Carvalho WS, Suffys PN, Kritski AL, Oliveira M, Zarade N, Zozio T, Rastogi N, Gicquel B (2011). Spoligotyping of clinical *Mycobacterium tuberculosis* isolates from the State of Minas Gerais. Brazil Mem Inst Oswaldo Cruz.

[67] Perizzolo PF, Dalla Costa ER, Ribeiro AW, Spies FS, Ribeiro MO, Dias CF, Unis G, Almeida da Silva P, Gomes HM, Suffys PN, Rossetti ML (2012). Characteristics of multidrug-resistant *Mycobacterium tuberculosis* in southern Brazil. Tuberculosis.

[68] Mendes NH, Melo FAF, Santos ACB, Pandolfi JRC, Almeida EA, Cardoso RF, Berghs H, David S, Faber KJ, Espanha LG, Leite SRA, Leite CQF (2011). Characterization of the genetic diversity of *Mycobacterium tuberculosis* in São Paulo city, Brazil. BMC Research Notes.

[69] de Oliveira MM, da Silva AR, Cardoso MO, Gomes HM, Fonseca L, Werneck-Barreto AM, Valim AM, Rossetti ML, Rossau R, Mijs W, Vanderborght B, Suffys P (2003). Rapid detection of resistance against rifampicin in isolates of *Mycobacterium tuberculosis* from Brazilian patients using a reverse-phase hybridization assay. J Microbiol Methods.

[70] Jardim PCR, Zamarioli LA, Coelho AGV, Figueiredo TR, Rozman MA (2001). Resistência do *Mycobacterium tuberculosis* às drogas no município de São Vicente. Rev Inst Adolfo Lutz.

[71] Filliol I, Motiwala AS, Cavatore M, Qi W, Hernando MH, Del Valle MB, Fyfe J, Garcia-Garcia L, Rastogi N, Sola C, Zozio T, Guerrero MI, Leon CI, Crabtree J, Angiuoli S, Eisenach KD, Durmaz R, Joloba ML, Rendon A, Sifuentes-Osornio J, de Leon AP, Cave MD, Fleischmann R, Whittam TS, Alland D (2006). Global phylogeny of *Mycobacterium tuberculosis* based on single nucleotide polymorphism (SNP) analysis: insights into tuberculosis evolution, phylogenetic accuracy of other DNA fingerprinting systems, and recommendations for a minimal standard SNP set. J Bacteriol.

[72] Veen J (1992). Microepidemics of tuberculosis: the stone-in-the-pond principle. Tubercle Lung Dis.

[73] Ferrazoli L, Palaci M, Marques LR, Jamal LF, Afiune JB, Chimara E, Martins MC, Silva Telles MA, Oliveira CA, Palhares MC, Spada DT, Riley LW (2000). Transmission of tuberculosis in an endemic urban setting in Brazil. Int J Tuberc Lung Dis.

[74] Narvskaya N, Mokrousov I, Otten T, Vyshnevskiy B, Read MM (2005). Molecular Markers: Application for Studies of *Mycobacterium tuberculosis* Population in Russia. Trends in DNA Fingerprinting Research.

